# Band reporting rates of waterfowl: does individual heterogeneity bias estimated survival rates?

**DOI:** 10.1002/ece3.791

**Published:** 2013-09-30

**Authors:** Gary C White, Line S Cordes, Todd W Arnold

**Affiliations:** 1 Department of Fish Wildlife and Conservation Biology, Colorado State UniversityFort Collins, CO 805325; 2 Department of Fisheries Wildlife and Conservation Biology, University of MinnesotaSt. Paul, MN 55108

**Keywords:** Band recoveries, bias, heterogeneous recovery rates, individual random effects, logit normal, ring recoveries, Seber model, waterfowl survival

## Abstract

In capture–recapture studies, the estimation accuracy of demographic parameters is essential to the efficacy of management of hunted animal populations. Dead recovery models based upon the reporting of rings or bands are often used for estimating survival of waterfowl and other harvested species. However, distance from the ringing site or condition of the bird may introduce substantial individual heterogeneity in the conditional band reporting rates (*r*), which could cause bias in estimated survival rates (*S*) or suggest nonexistent individual heterogeneity in *S*. To explore these hypotheses, we ran two sets of simulations (*n* = 1000) in MARK using Seber's dead recovery model, allowing time variation on both *S* and *r*. This included a series of heterogeneity models, allowing substantial variation on logit(*r*), and control models with no heterogeneity. We conducted simulations using two different values of *S*: *S* = 0.60, which would be typical of dabbling ducks such as mallards (*Anas platyrhynchos*), and *S* = 0.80, which would be more typical of sea ducks or geese. We chose a mean reporting rate on the logit scale of −1.9459 with SD = 1.5 for the heterogeneity models (producing a back-transformed mean of 0.196 with SD = 0.196, median = 0.125) and a constant reporting rate for the control models of 0.196. Within these sets of simulations, estimation models where σ_S_ = 0 and σ_S_ > 0 (σ_S_ is SD of individual survival rates on the logit scale) were incorporated to investigate whether real heterogeneity in *r* would induce apparent individual heterogeneity in *S*. Models where σ_S_ = 0 were selected approximately 91% of the time over models where σ_S_ > 0. Simulation results showed < 0.05% relative bias in estimating survival rates except for models estimating σ_S_ > 0 when true *S* = 0.8, where relative bias was a modest 0.5%. These results indicate that considerable variation in reporting rates does not cause major bias in estimated survival rates of waterfowl, further highlighting the robust nature of dead recovery models that are being used for the management of harvested species.

## Introduction

Management of North American waterfowl relies heavily on survival rates (*S*) estimated from band recovery models (Brownie et al. 1985). Waterfowl are banded annually, primarily during late summer on the breeding grounds, and bands are recovered from hunters that harvest birds during the ensuing hunting season. Models used for estimating survival rates assume that there is no individual heterogeneity of the conditional band reporting rates, *r*. Seber (1970) defined band reporting rates (*r*) conditional on the bird dying, 1−*S*, so that the probability a band is reported is (1−*S*)*r*, which is equivalent to the band recovery rate (*f*) of Brownie et al. (1985).

Individual heterogeneity in *r* might occur for a myriad of reasons, including differential survival between time of banding and onset of hunting (Nichols et al. 1982; Zimmerman et al. 2010), differential vulnerability to harvest (Pollock and Raveling 1982; Pace and Afton 1999), or differential probability that hunters will report a recovered band (Henny and Burnham 1976; Reinecke et al. 1992). However, detection of individual heterogeneity in conditional band reporting rates is difficult, if not nearly impossible. The estimate of *r* is a purely binomial process, with each bird that dies having only a Bernoulli trial as to whether its band is reported. That is, a bird only dies once, and so there cannot be repeated trials of whether a band is reported that would allow detection of individual heterogeneity. In contrast, repeated trials of birds surviving annual intervals in a band reporting analysis provide the necessary information to detect individual heterogeneity in survival rates, and hence, estimation of σ_*S*_ in the models described below, and repeated recapture or resighting of living birds in Cormack–Jolly–Seber models allows for the detection of individual heterogeneity in detection probability (*P*).

One approach that might be used to detect individual heterogeneity of conditional band reporting rates would be to separate birds into independent analyses and then compare the estimates of *r* across these lots, in the sense of Burnham et al. (1987), Part 4. However, this approach is generally inefficient and would be unlikely to detect low amounts of heterogeneity. A second approach is the use of covariates or groups (Dorazio 1997). However, only covariates measured at the time of banding are available, because potential covariates at the time of recovery are only available for reported bands. Further, these covariates have to correlate with the conditional band reporting rate (*r*) which seems unrealistic except for a few easy to measure covariates such as age or body mass at time of banding (Pace and Afton 1999). Thus, the covariate approach is again not likely to provide much explanatory power for detecting individual heterogeneity of *r*.

Even if individual heterogeneity in *r* can be shown, the real issues are whether this effect causes bias in the estimates of survival and whether individual heterogeneity in *r* will result in the detection of nonexistent individual heterogeneity in *S*. Therefore, our objective was to evaluate the potential for incorrect inference about individual heterogeneity of *S* and the potential for biased estimates of *S* when considerable individual heterogeneity exists in *r*.

## Methods

**Figure 1:**
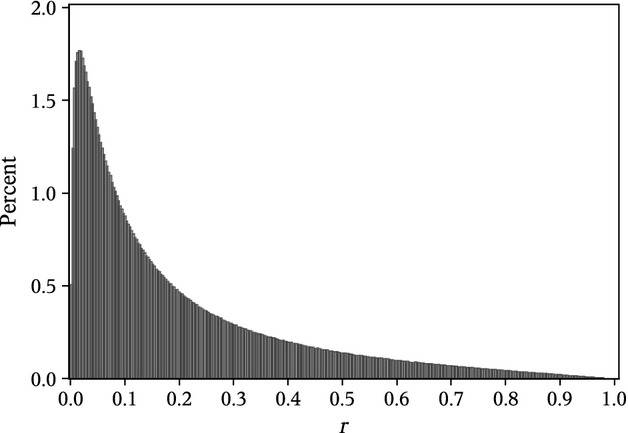
Distribution of the back-transformed value logit(*r*) ∼ *N*(−1.9459, 1.5) used in our simulations, giving mean of 0.196 with SD = 0.196 and median = 0.125. Note that log[0.125/(1−0.125)] = −1.9459

Sixteen sets of simulations of Seber's (1970) single age class ring recovery model, each with 1000 replicates, were conducted as a factorial design. Four factors, each with 2 levels, were considered: (1) the focal effect of interest of identical or else individual variation in band reporting rate (*r*), (2) constant band reporting rate *r*(.) or time-specific band reporting rates *r*(*t*), (3) low 0.6 or high 0.8 levels of annual survival, and (4) annual survival assumed constant *S*(.) or time-specific *S*(*t*). Thus, half the simulations had constant reporting rate (*r*) of 0.196 for each individual, with the other half with a reporting rate drawn on the logit scale from a normal distribution with mean = −1.9459 with SD = 1.5, giving the random reporting rate with a back-transformed mean of 0.196 with SD = 0.196 and median = 0.125 (Fig. 1). Although time-specific effects were assumed for *S* and *r* in half of the analyses, actual values simulated were identical across time in all cases. Thus, only 4 different scenarios of data generation were performed, but with 4 estimation models applied to each scenario. For estimation model *S*(*t*),*r*(.) with time-specific 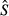 and constant *r*, the mean of the 10 survival estimates was used as the overall estimate of 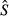, and for model *S*(*t*),*r*(*t*), the mean of the first nine survival estimates was used. All simulations were conducted with constant annual survival rates, that is, there was no temporal or individual heterogeneity in survival. Ten occasions were simulated, with 10,000 birds assumed banded and released on each occasion to provide high statistical power for selecting among models.

For each of the 16 sets of simulations, 2 models were estimated. The first modeled no individual variation in survival, that is, all individuals were assumed to have identical survival rates (σ_*S*_ = 0). The second more complex model assumed individual heterogeneity in survival (σ_*S*_ estimated) modeled as an individual random effect on the logit scale, following the approach of McClintock and White (2009), McClintock et al. (2009), and Gimenez and Choquet (2010). That is, for the *i*^th^ individual, logit(*S*_*i*_) = μ_*S*_ + *z*_*i*_, where *z*_*i*_ ∼ N(0, σ_*S*_). The parameter σ_*S*_ is estimated via maximum likelihood using numerical integration (Gaussian–Hermite quadrature, Givens and Hoeting 2005) to solve the integral numerically. For models assuming time-specific survival, a single parameter σ_*S*_ is estimated that applies to all of the survival estimates, that is, all the time-specific survival estimates for individual *i* have the same *z*_*i*_ on the logit scale. For each replicate, these 2 models were compared using AIC_*c*_ to determine the selected model and the relative weight of each of the 2 models. There is 1 df of difference between the 2 models, depending on whether σ_*S*_ is estimated or assumed equal to zero; hence, models with σ_*S*_ > 0 can appear competitive even though σ_*S*_ = 0 (Burnham and Anderson 2002:131). All simulations and parameter estimates were produced using Program MARK (White and Burnham 1999).

## Results

Overall, models with σ_*S*_ estimated were selected 8.9% of the time (Table 1). There was some evidence that rate of selection for model σ_*S*_ differed between data sets simulated with or without individual heterogeneity in conditional band reporting rates (i.e., σ_*r*_ = 1.5 or 0 on the logit scale), but the selection rate was higher for data sets with σ_*r*_ = 1.5 when survival was 0.6, but lower for data sets with σ_*r*_ = 1.5 when *S* was 0.8 (Table 2; G_adj_ = 10.68, df = 3, *P* = 0.01). In addition, σ_*S*_ was selected more frequently when it was included in model structures that included temporal variation in both *S* and *r* (Table 2; G_adj_ = 63.57, df = 3, *P* < 0.0001).

**Table 1:** Model selection results summarized for the model assuming individual heterogeneity in survival (σ_*S*_ estimated) or no heterogeneity (σ_*S*_ = 0) by whether there is individual heterogeneity in the conditional band reporting rate (σ_*r*_ = 1.5 on logit scale) or not (σ_*r*_ = 0). Values are the number of times a model was selected (percent of times selected) for each of the four combinations

	Times selected		
		
True σ_*r*_	σ_*S*_ estimated (%)	σ_*S*_ = 0 (%)	Total
1.5	698 (8.7)	7302 (91.3)	8000
0	719 (9.0)	7281 (91.0)	8000
Total	1417 (8.9)	14,583 (91.1)	16,000

**Table 2:** Percent of times out of 1000 simulations that models with σ_*S*_ estimated ranked higher than models with no heterogeneity (σ_*S*_ = 0) depending on true survival rate (0.6 or 0.8), whether there was individual heterogeneity in the conditional band reporting rate (σ_*r*_ = 1.5 or 0.0 on logit scale), and whether or not the estimating models included temporal variation in *S*(*t*) or *r*(*t*)

		Percent times σ_*S*_ > 0 selected over σ_*S*_ = 0				
			
True *S*	True σ_*r*_	*S*(.), *r*(.)	*S*(.), *r*(*t*)	*S*(*t*), *r*(.)	*S*(*t*), *r*(*t*)	Total
Low 0.6	1.5	9.3	7.4	7.8	10.2	8.7
	0	7.4	7.4	6.6	10.5	8.0
High 0.8	1.5	7.7	8.1	7.1	12.2	8.8
	0	7.6	7.4	9.8	15.3	10.0
Total		8.0	7.8	7.8	12.1	8.9

Note that because there is only a single degree of freedom difference between models with and without σ_*S*_, the expected difference between the −2log likelihood of the 2 models would be a χ^2^ random variable with 1 degree of freedom. Thus, the model with σ_*S*_ estimated would only be selected when the −2log likelihood of the σ_*S*_ = 0 model is 2 units larger (given that the effective sample size of these simulations is large, and AIC_*c*_ and AIC are nearly identical). The Pr(χ^2^_(1)_ > 2) = 0.157, suggesting that the more complex model should have been selected 15.7% of the time, but our simulations show that in all cases, the more complex model is selected less often. However, the null hypothesis σ_*S*_ = 0 is on the boundary of the parameter space, and therefore, classical inference as described above no longer holds (Self and Liang 1987; Gimenez and Choquet 2010). The asymptotic null distribution of the LRT is a 50:50 mixture of χ^2^ distributions with 0 and 1 degrees of freedom (Stram and Lee 1994). Thus, our simulations are consistent with this theory in that models with σ_*S*_ estimated were selected just over half (8.9 vs. 15.7) of what is expected.

Average model weights were consistent across the levels of survival and whether or not individual heterogeneity is present in the conditional band reporting rate (Table 3). Given that the theoretical expected difference in the −2log likelihood value between the 2 models is χ^2^_(1)_, the expected ΔAIC is 1. Given this expected ΔAIC, the expected weight of the more complex model would be 0.378. However, as described above, this theory does not hold, so we would expect our simulations to show better performance than expected by assuming the difference to be χ^2^_(1)_.

**Table 3:** Model weight results summarized for the model assuming individual heterogeneity in survival (σ_*S*_ estimated) or no heterogeneity (σ_*S*_ = 0) by two levels of survival and whether there is individual heterogeneity in the conditional band reporting rate (σ_*r*_ = 1.5 on logit scale) or not (σ_*r*_ = 0). Values are the average model weight based on 4000 simulations for each scenario, with the SE of all entries ≤0.002

		Average model weights	
		
True *S*	True σ_*r*_	σ_*S*_ estimated	σ_*S*_ = 0
Low 0.6	1.5	0.330	0.670
	0	0.326	0.674
High 0.8	1.5	0.330	0.670
	0	0.333	0.667
Average		0.330	0.670

Our simulations suggest little bias in 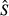 between data sets that include individual variation in *r* (σ_*r*_ = 1.5 on the logit scale) versus those that do not (σ_*r*_ = 0) (Table 4). Average percent bias, 100 × (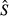 − *S*)/*S,* was less than 0.1% for all estimation models when true *S* = 0.6, with all means accurate to 3 decimal places (Table 4). When true survival was 0.8, models that estimated individual heterogeneity in survival exhibited positive relative bias of 0.5%, but bias was not exacerbated by heterogeneity in conditional band reporting rates. Relative to the expected CV of waterfowl survival estimates (mean 6.8% over 32 studies), this percent relative bias appears insignificant. These results indicate that considerable variation in reporting rates does not cause appreciable bias in estimated survival rates of waterfowl, further highlighting the robust nature of dead recovery models that are being used for the management of harvested species. Further, the SD of 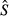 was only slightly different between scenarios estimating σ_*S*_ versus those with σ_*S*_ = 0 (Table 4).

**Table 4:** Mean and SD of survival estimates and percent relative bias in 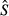 under 16 data simulating models, each analyzed with two different estimating models (σ_*S*_ estimated, or σ_*S*_ = 0). Survival estimates were not affected by whether or not temporal variation in *S* or *r* was estimated, so results are collapsed. For estimation model *S*(*t*),*r*(.) with time-specific 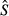 and constant *r*, the mean of the 10 survival estimates was used as the overall estimate of 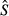, and for model *S*(*t*),*r*(*t*), the mean of the first nine survival estimates was used. Values are the average estimated survival based on 4000 simulations for each scenario, all with SE <0.0005

		Estimation model					
		
		σ_*S*_ estimated			σ_*S*_ = 0		
			
True *S*	True σ_*r*_	Mean	Bias (%)	SD	Mean	Bias (%)	SD
0.6	1.5	0.6000	0.00	0.0038	0.6000	0.00	0.0037
	0	0.6003	0.05	0.0038	0.6002	0.04	0.0038
0.8	1.5	0.8040	0.50	0.0076	0.8002	0.02	0.0045
	0	0.8042	0.53	0.0077	0.8002	0.03	0.0045

**Figure 2:**
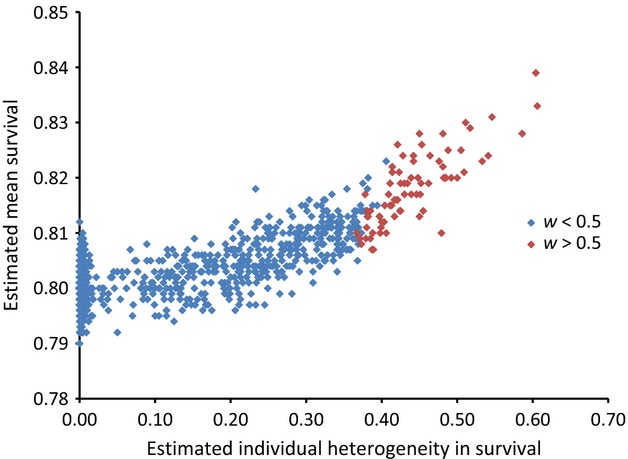
Relationship between estimated individual heterogeneity in survival and estimated mean survival rate for 1000 simulations where true *S* = 0.8, σ_*S*_ = 0, *r* = 0.196, and σ_*r*_ = 1.5. Estimation models were S(.),r(.) with σ_*S*_ estimated or fixed to zero. Blue data points indicate 923 data sets where the simpler model lacking σ_*S*_ was selected by AIC_*c*_ (*w*_*i*_ of model [σ_*S*_, S(.),r(.)] < 0.5), whereas red dots indicate 77 data sets where model σ_*S*_ was selected (*w*_*i*_ > 0.5).

Lastly, 1417 times out of 16,000 simulations (8.9%), the model that estimated σ_*S*_ would have provided an estimate. These estimates (Table 5) will always be >0 because σ_*S*_ is estimated on the log scale to maintain this constraint. As expected, for simulations with *S* = 0.8 and σ_*S*_ estimated, 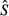 − *S* was positively correlated with 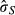 (Fig. 2). The likelihood of selecting the minimum AIC_*c*_ model that estimates σ_*S*_ is not independent of the true *S* and the true σ_*r*_: χ^2^_(1)_ = 4.064, *P* = 0.044. The estimated σ_*S*_ is larger for true *S* = 0.8 than for *S* = 0.6, which makes sense because σ_*S*_ is on the logit scale. Had models where σ_*S*_ is estimated only been considered, the expected values again would be >0 because σ_*S*_ is estimated on the log scale to maintain this constraint (Table 6). However, in comparing the values in Table 5 with values in Table 6, we see that the expected value is nearer zero. We also see minor difference in Table 6 of the mean values of 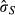 for simulations with σ_*r*_ > 0 versus σ_*r*_ = 0.

**Table 5:** Estimates of σ_*S*_ when the models producing this estimate were selected by minimum AIC_*c*_

True *S*	True σ_*r*_	*N*	Mean	Std. Error	Minimum	Maximum
0.6	1.5	347	0.254	0.0017	0.195	0.380
	0	319	0.247	0.0015	0.195	0.382
0.8	1.5	351	0.432	0.0039	0.273	0.638
	0	400	0.429	0.0037	0.143	0.671

**Table 6:** Estimates of σ_*S*_ from all simulations for models producing this estimate

True *S*	True σ_*r*_	Mean	Std. Error	Minimum	Maximum
0.6	1.5	0.0804	0.00142	0	0.380
	0	0.0774	0.00140	0	0.382
0.8	1.5	0.1377	0.00244	0	0.638
	0	0.1412	0.00248	0	0.671

## Discussion

As with any simulation study, and particularly one with the limited parameter space considered in this study, care must be taken in drawing inferences to other sets of parameter values. Our parameter values were focused on waterfowl because this is the primary assemblage of species where these models are used for management decisions. However, we are encouraged by how little effect individual heterogeneity in *r* had in causing the analyst to conclude that there was individual heterogeneity in *S* compared with the same error rate for data with no heterogeneity in *r*. But as shown in Table 5, these error rates differ depending on the magnitude of *S*, although the number of birds banded and mean *r* were constant. A wider range of parameter values, and probably more importantly, a wide range of number of birds banded and values of *r*, would be needed to evaluate what the error rate is in selecting the model estimating σ_*S*_ over the model with σ_*S*_ = 0, and how this error rate is affected. All simulations were conducted under the artificial scenario of no individual variation in *S*. Simulations encompassing a reasonable range of individual variation in *S* would be helpful, but were beyond the scope of this article.

Pollock and Raveling (1982) first claimed that heterogeneous band recovery rates would not bias estimates of survival rates, and our simulations further confirmed this result. The only scenarios where we found appreciable bias was when survival was high and models estimated individual heterogeneity in survival, even though data were simulated with σ_*S*_ = 0. However, there is undoubtedly real individual heterogeneity in *S* in most natural populations, for example Rexstad and Anderson (1992), and this individual heterogeneity is likely not always picked up by the use of covariates or group attributes as advanced by Dorazio (1997). Thus, assessing individual variation in *S* through the use of the individual heterogeneity model considered here should be routinely undertaken by managers. However, we advise caution, especially when survival rates are high, because overestimation of 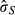 can lead to overestimation of 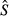. And we still encourage the collection of individual covariates at the time of banding, if only to compare these models against the models with σ_*S*_ estimated. Covariates attempt to explain the causes of individual heterogeneity, rather than just accommodate the heterogeneity as with models estimating σ_*S*_.

Pollock and Raveling (1982) did find that heterogeneity in band recovery (*f*) or conditional reporting rates (*r*) could lead to bias if it was accompanied by individual heterogeneity in survival, especially if σ_*S*_ was correlated with σ_*f*_ (see also Nichols et al. 1982; Barker 1992). Such correlation might be expected if some individuals are more vulnerable to hunting (thus, higher *f* or *r*), and such vulnerability results in (or is correlated with) lower survival (Nichols et al. 1982; Sedinger and Herzog 2012). We did not consider individual heterogeneity in survival in our simulations, so analysts should beware of applying our results in cases where heterogeneity in conditional reporting rate might be correlated with heterogeneity in survival rates, and we recommend additional simulation studies to explore this condition.

There are likely two main sources of individual variation in conditional reporting rates of hunted waterfowl: (1) individual variation in vulnerability to hunting (Pace and Afton 1999; Arnold and Howerter 2012) and (2) individual variation in the probability that a recovered band will be reported to authorities (Henny and Burnham 1976, Royle and Garrettson 2005; Zimmerman et al. 2009). Our results are most relevant to the second scenario, as it is difficult to imagine how reporting rates would be correlated with survival. In North America, band inscriptions changed twice during 1993–1995, with the inclusion of zip codes in 1993 and toll-free phone numbers in 1995, and these changes have been considered problematic by analysts analyzing band recovery data during this time period (Royle and Garrettson 2005). However, our results suggest that mean survival rates and indeed mean band reporting rates are unlikely to be biased by heterogeneity in reporting rates caused by changing band inscriptions. Other studies have noted regional variation in band reporting rates (Zimmerman et al. 2009), and so long as the management goal is to estimate overall survival rate for a population, such heterogeneity is unlikely to lead to biases in survival estimation.
